# EdTech for Ugandan girls: Affordances of different technologies for girls' secondary education during the Covid‐19 pandemic

**DOI:** 10.1111/dpr.12619

**Published:** 2022-09-05

**Authors:** Kalifa Damani, Rebecca Daltry, Katy Jordan, Libby Hills, Laura Evans

**Affiliations:** ^1^ EdTech Hub ‐ Jigsaw Consult London UK; ^2^ Jigsaw Consult London UK; ^3^ Faculty of Education University of Cambridge UK; ^4^ Promoting Education in African Schools London UK

**Keywords:** Covid‐19, EdTech, education, gender, rural Uganda

## Abstract

**Motivation:**

This article discusses the use of educational technology (EdTech) in girls' education at PEAS (Promoting Education in African Schools) schools in rural Uganda during the Covid‐19‐related school closures.

**Purpose:**

This article addresses a research gap surrounding the potential use of EdTech to support girls' education, focusing on the barriers to girls' EdTech use and how technology might be used to enhance girls' education in disadvantaged rural areas—specifically their academic learning and their social and emotional learning.

**Methods and approach:**

A sequential, explanatory mixed‐methods case‐study approach was used. Quantitative exploration of a dataset of 483 Ugandan students, from 28 PEAS schools, was first conducted, followed by interviews with PEAS staff to elucidate the reasons and context behind the findings.

**Findings:**

Findings show that female students are less likely than male students to have access to their caregivers' phones for learning. The form of EdTech that appeared to be most beneficial for girls' academic learning was radio; girls also had significantly more interest in tuning into radio broadcasts than boys did. Also, poorer boys were more likely to be influenced by SMS messages than wealthier boys. Apart from gender‐based differences, students with more highly educated parents found SMS messages more helpful, and phone calls from teachers appeared to help boost younger students' self‐confidence.

**Policy implications:**

The findings suggest that policy‐makers need to: carefully consider provision of education through multiple modes of EdTech in order to ensure that it reaches all students; ensure that caregivers are involved in the strategies developed for girls' education; make EdTech interventions interactive; and consider language in EdTech interventions. Given the gender differences which emerged, the findings are of relevance both to supporting the continuation of educational provision during periods of school closure, and also in terms of finding additional ways to support girls' education alongside formal schooling.

## 1 INTRODUCTION

Inequality in access to quality education for girls is a significant global challenge. Addressing this has the potential to bring a range of social benefits, from personal empowerment of girls and women to economic growth and advancing the sustainable development goals (Sperling & Winthrop, 2016). As such, girls' education is a priority for many international development organizations, reflected in programmes such as the UK Foreign, Commonwealth and Development Office (FCDO) Girls' Education Challenge launched in 2012, and reaffirmed in the newly published FCDO five‐year action plan (FCDO, 2021).

While girls' access to education has increased, this is likely to have slowed as a result of the Covid‐19 pandemic. During school closures, the use of technology has played an integral role in many countries' pandemic response to continuing educational provision, from radio and television broadcasts to dedicated online platforms (Dreesen et al., 2020). Such use of technology has also been an important tool for ensuring continued access to education in times of crises over the past decade (Dhawan, 2020). Radio, for example, was used for children’s continued literacy, numeracy, health and self‐esteem education during the Ebola epidemic in Sierra Leone (Barnett et al., 2018) and social media and e‐learning for university students in New Zealand after seismic events (Tull et al., 2017). Educational technology (EdTech) encompasses a wide range of digital and non‐digital technologies designed or appropriated for use in educational contexts (Hennessy et al., 2021). Previous research has shown that the use of EdTech to support girls’ education specifically can bring both benefits and challenges, and needs to be contextually appropriate (Girls Education Challenge, 2018). Furthermore, the use of EdTech risks deepening educational divides in relation to other factors, such as urban and rural locations, where levels of access to technology and connectivity are lower (UNICEF, 2021). While the impact of the pandemic is yet to be fully understood and documented, some evidence is emerging to suggest that it may have amplified inequalities relating to existing gender divides in access to technology. Data from Kenya show that girls have experienced a greater degree of “learning loss” during the pandemic than boys and there is a gap between rural and urban learners (Whizz Education, 2021). Experience of previous crises suggests that girls may be less likely to return to school following closures (Hallgarten, 2020).

Despite the focus upon girls' education in recent years, the research literature exploring the potential role for EdTech is limited, and questions remain regarding how transferable the findings are from existing studies to other contexts. There is a need for further research into the use of EdTech for furthering girls' academic progress and social and emotional learning (SEL). Additionally, there is a need for research that focuses on reaching specifically marginalized girls in rural areas who potentially face the greatest barriers during periods of school closure.

This article aims to help narrow those gaps, by focusing on how technology might be used to enhance girls' education. It presents an empirical analysis of a recent survey dataset, which was collected at 28 PEAS (Promoting Education in African Schools) schools in Uganda in November 2020. PEAS schools focus upon education in rural and low‐income communities within Uganda; by focusing on this context, insights are provided into reaching the most marginalized across the education system. Special consideration is given to the additional challenges raised by Covid‐19 and distance learning. The following research questions are addressed by the study:

1. Do challenges of using different types of EdTech (SMS messaging, telephone calls, and radio) vary significantly by gender?
2. What are the mediating factors (poverty, gender, parents' education, age, chores) that might be related to finding different EdTech (SMS messaging, telephone calls, and radio) helpful or motivating?
3. Which mode of EdTech is the most beneficial for girls' and boys' learning at home, and why is this the case?
4. Which mode of EdTech (SMS messaging, telephone calls, and radio) is the most beneficial for girls' and boys' self‐esteem and self‐confidence development, and why is this the case?

First, we outline the background to the study, including reviewing the existing research literature in relation to girls' education and EdTech in low‐income, lower‐middle income and upper‐middle‐income countries (LMICs), and introduce the context for this study. Section 3 sets out the methodology, will detailing the research design, data collection, and analysis used for the empirical part of the study. Results are then presented in full in Section 4, and Section 5 summarizes the implications of the findings and future research directions.

## 2 BACKGROUND AND CONTEXT

The existing research literature on girls' education and EdTech suggests a relationship which is complex. There is a tension within the literature; while some evidence suggests that EdTech can be particularly beneficial for girls and help to close gender gaps, sociocultural factors may mean that girls are less likely to be allowed access to technology. However, the evidence base is limited, and further research—from a wider range of contexts—is needed. In this section, we will first discuss the existing literature in relation to this tension, before focusing upon the particular context for the study.

The few existing research studies focusing on girls' education and EdTech within LMICs suggest that girls' and womens' usage of technology can be “disproportionately more empowering for girls relative to boys, with wider benefits which expand beyond formal education” (Webb et al., 2020, p. 6). For example, female users of the Worldreader app—a platform providing e‐books to children and their families across 11 sub‐Saharan African countries—were found to make significantly greater use of the resource than male readers (West & Chew, 2014). A recent evaluation of personalized learning software in Malawi—where substantial gender gaps persist—showed significant learning gains in relation to reading and numeracy for girls, suggesting that the use of the software can help prevent gaps from emerging (Pitchford et al., 2019).

However, there is a major lack of robust research evidence in relation to EdTech use in LMICs, which specifically addresses the impacts in terms of gender. The existing evidence base was recently expanded retrospectively; Evans and Yuan (2021) revisited and updated interventions from a previous systematic review, contacting authors of the original studies in order to request the findings disaggregated by gender. EdTech examples can be found at both extremes in relation to girls' learning outcomes; hardware alone can have a negative effect, while some software interventions are among the top ranked studies (Evans & Yuan, 2021). The limited number of EdTech studies within the review illustrates that there is a need for more robust evidence in relation to EdTech use in LMICs in general. Furthermore, the range of studies included in the reviews focus on learning outcomes in terms of literacy and numeracy, while other outcomes such as SEL remain underexamined (RTI International, 2018).

While EdTech can be beneficial to girls' education, there is a key concern that the use of technology will further gender segregation unless a project attends to the “gendered nature of human interactions with technology” (Steeves & Kwami, 2017, p. 184). Choosing contextually relevant digital “solutions” and attending to men’s attitudes towards women and EdTech are important considerations for governments seeking to implement technology initiatives for girls’ education (Unwin et al., 2021).

The gendered digital divide appears most evident outside of school (Webb et al., 2020). Several factors contribute, including gendered household roles and attitudes, cost of technology, fears surrounding security and control over access, and mobility. Multiple studies highlight greater freedom for boys to use computers, for leisure or study, outside of school, with examples reported from Rwanda (Rubagiza et al., 2011), Central African Republic and Congo (Yeba, 2012), India (Basavaraja & Sampath Kumar, 2017) and Ghana (Steeves & Kwami, 2017). Furthermore, girls were found to have less time and disposable income to engage with technology, due to household responsibilities (Rubagiza et al., 2011; Steeves & Kwami, 2017; Zelezny‐Green, 2018). Zelezny‐Green (2011), for example, highlights the way in which the ownership and usage of technology are framed as “masculine” in Kenya. Vilakati (2014) and Yeba (2012) show how this attitudinal bias is reinforced by girls' parents, teachers, and the students themselves, in Eswatini (Vilakati, 2014) and Cameroon, Central African Republic, and Congo (Yeba, 2012). However, such perceptions may differ in practice, and be changing over time. Chuma (2014) notes that young women students in South Africa use their mobile phones to a greater extent—in terms of both time and purposes—than their male counterparts, while a recent study by Porter et al. (2020) highlights several ways in which increased mobile phone ownership among youth in sub‐Saharan Africa has empowered young women.

Girls are also less likely to access shared community facilities due to sociocultural, gendered norms. Girls' use of cyber cafés may be actively discouraged: Yeba (2012) noted the negative public opinion towards girls who use cafés in central Africa, while Steeves and Kwami (2017) described how girls in Ghana were sometimes not even permitted entry. This also underlines the importance of providing access to safe spaces—whether physical or virtual—and attending to safeguarding considerations when providing internet and technology for girls (Naylor & Gorgen, 2020). There is a substantial body of research evidence from contexts across the globe which illustrates the potential risks to children online, including cyber‐bullying and sexual exploitation, which are often experienced disproportionately by girls (Stoilova et al., 2021).

The literature identifies gatekeepers to girls' access to technology, and that the gender digital divide may be exacerbated through use of EdTech; “unless parents and teachers are involved in programme development and receive adequate and ongoing training in technology usage and gender‐responsive teaching” (Webb et al., 2020, p. 6). Girls themselves may also “self‐regulate,” exhibiting cultural values and beliefs towards their use of technology. Yeba (2012) noted a feeling of “technophobia,” with girls afraid of breaking hardware, while Zelezny‐Green (2014) observed reluctance to use mobile phones in Kenya, due to reservations about inappropriate usage.

This also points to a more general issue that gender norms and access may also differ according to the type of technology involved, so it may be useful for research to consider a range of EdTech beyond computers or tablets, to include cheaper and accessible devices (for example, radios or mobile phones) (Damani & Mitchell, 2020; Webb et al., 2020). Zelezny‐Green’s work in Kenya (2014; 2018) indicates that girls increasingly have independent access to mobile phones, and accessing educational content this way could mediate interrupted school attendance. While this reflects a broader trend of increasing mobile phone access and ownership by children and youths at a global level, gender gaps are observed (Girl Effect & Vodafone Foundation, 2018; Tyers‐Chowdhury & Binder, 2021). This is particularly pertinent to the ongoing impact of the Covid‐19 pandemic. Similarly, radio—especially interactive radio instruction (IRI) approaches—can deliver educational content to hard‐to‐reach students in a relatively cost‐effective manner (Damani & Mitchell, 2020). A small number of studies have considered the gender‐related advantages of this medium. Nekatibeb and Tilson (2004) identified that IRI increased learning gains in Ethiopian primary schools, noting that female students learned more through IRI than their male counterparts. Similarly, the Pikin to Pikin Tok programme—which centred on educational messaging via radio for behavioural change, particularly targeting health practices in the context of the Ebola crisis in Sierra Leone—was found to increase community prioritization of girls’ education (Barnett et al., 2018). Again, this suggests that this medium could provide girls with continued access to education in emergency contexts.

While there is acknowledgement that in many countries girls are less likely to have access to technology, it is challenging to quantify the extent of this disadvantage, as these data are rarely available for children and adolescents (Tyers‐Chowdhury & Binder, 2021). Similarly, definitive evidence is lacking in terms of the impact of reduced technology access upon learning outcomes, although it was suggested that female students experience less equitable access to EdTech resources in the classroom, illustrated by examples from Rwanda (Rubagiza et al., 2011), Central African Republic, and Congo (Yeba, 2012). This may be due to existing gendered assumptions: teachers' low expectations towards girls' competence and enjoyment of technology were identified in an example of technology‐based personalized learning to prevent gender gaps in Malawi (Pitchford et al., 2019), while girls' preferences not to attend computer labs were evidenced in Rwanda (Rubagiza et al., 2011).

The landscape of existing literature includes promising examples of the potential for EdTech to be used to promote positive learning outcomes for girls in LMICs. However, sociocultural factors in relation to gender and the use of technology risk exacerbating educational divides. This underscores the importance of focusing upon context, and the need for further research to help untangle issues in relation to gender, different forms of technology, and its use in education.

### 2.1 Context: Girls' education and EdTech in Uganda

This article focuses specifically upon secondary school girls' engagement with EdTech in Uganda. Across Uganda, female enrolment tends to decrease as grade levels increase; 47.66% of students enrolled in grade S4, 41.08% in S5, and 39.71% in S6 are female (MoES, 2017). There is a persistent gender gap in relation to educational attainment, which under current projections is unlikely to close until 2038 (Evans et al., 2020). Additionally, as of 2017, 24.6% of secondary school teachers were female (MoES, 2017), which may impede efforts to implement gender‐responsive teaching through technology (Okudi, 2016).

The Government of Uganda has also initiated various projects for technology‐enhanced learning over the past decade, including the Connect‐ED and CurriculumNet projects, and Information and Communication Technology (ICT) has been one of the fastest growing sectors in the country since 2010 (Barakabitze et al., 2019). The national framework for ICT policy, Digital Uganda Vision, seeks to provide “a unified direction for ICT development and an Integrated ICT project implementation approach” in various fields, including education (Ministry of ICT & National Guidance, n.d.).

While the potential for EdTech to be used to support education in Uganda has been identified, there is currently a lack of research into its use to support school‐level learners at all, let alone considering how this may be experienced by different genders. Most of the literature found through the search on EdTech in Uganda focused on its implementation in tertiary education. These studies mainly examine the factors which facilitate and hinder ICT use in distance learning, content delivery, university administration, and teacher training (Ali et al., 2013; Habibu et al., 2012; Matovu, 2012; Oroma et al., 2013; Zhu & Mugenyi, 2015).

In relation to school‐based education, there are notable examples of projects which have often used messaging‐based technologies. For example, the MobiLiteracy Uganda Program (Pouezevara & King, 2014) sought to enhance literacy in primary school pupils (Grades 1 and 2) through audio content and SMS support. Twaweza’s two programmes, Uwezo and Sauti za Wananchi (cited in Koomar & Blest, 2020), also used this platform to conduct an annual citizen‐led assessment of children’s learning levels and a nationally representative interactive voice response (IVR) mobile phone survey, respectively.

In addition to the need to address gender‐based gaps in basic education, the importance of SEL for girls' development—as a way to help address persistent gender gaps in education more generally, and as preparation for skills required following school—is acknowledged in Uganda (Malhotra, et al., 2021; MoES, 2013). However, the potential use of EdTech to support this in Uganda remains unexplored in the research literature at present.

While highlighting these opportunities, the literature also notes the potential limitations of the country’s current ICT levels. Although mobile cellular subscriptions increased between 2015–2019, Uganda had an active mobile subscription rate of 34 per 100 inhabitants in 2019 (just above the African average of 33.1, but well beneath the world average of 75) (ITU, 2021). Only 11% of households were found to have internet access, perhaps impacted by the major constraint of limited electricity rates across the country (ITU, 2021). When schools were closed in March 2020 to prevent the spread of the Covid‐19 virus, the government turned to low‐tech strategies for the continuation of education provision at home, including home learning packs, and lessons broadcast by radio and television (Center for Global Development, 2021; Kyamazima, 2020).

### 2.2 Introduction to PEAS

PEAS, a non‐governmental organization (NGO), has been operating in Uganda since 2008, and provides the framework for the empirical work in this study. Its remit is to expand access to quality secondary education in rural and low‐income communities across Africa, building and running secondary schools where children previously had limited education options after finishing primary school. According to their website and interviews with staff members, PEAS has educated over 30,000 marginalized students since 2008 and currently runs 28 non‐state secondary schools across Uganda (PEAS, n.d.). PEAS schools are co‐educational but, due to the additional challenges girls face (such as early pregnancy which leads to girls dropping out of school, lack of gender sensitive sanitation facilities, or gender bias and inequitable attitudes, which can discourage girls from participating in school and limit the support and encouragement they receive), the NGO places a particular focus on supporting girls to enrol in secondary school and successfully complete their education, and helping prepare them for life after school (PEAS, n.d.).

On March 20, 2020, the Government of Uganda closed all schools to prevent the spread of Covid‐19 (Kyamazima, 2020). According to PEAS, only a minority of PEAS households have access to the internet, computers, and smartphones, but the majority have basic mobile phones and access to radio. PEAS therefore designed a package of no‐ and low‐tech remote learning interventions intended to keep students (especially girls) safe, engaged, and educated, and increase the likelihood that they returned once schools reopened. PEAS' remote learning programme was implemented throughout 2020, with a more streamlined version continuing into 2021 as schools in Uganda slowly reopened.

PEAS' remote learning programme had three main EdTech components—telephone calls (known within the programme as a “telephone tree system,” which involved teachers making phone calls to students), a programme of SMS text messages, and radio lessons. Note that access to telephones at home are likely to have been mediated by gatekeepers, such as parents and other older household members. Participation in the programme was not compulsory. At the time that the survey took place the SMS programme was one‐way: PEAS sent out SMS messages to caregivers and students, but they were not expected to respond to the messages. Text messages shared important information, such as health and safety guidance. They also focused on positive wellbeing as well as academic content.

PEAS developed a telephone call guide to provide teachers with a clear framework for making support calls, organized into four stages: Connect, Protect, Inform and Educate:

- Connect—build rapport with the parent and student
- Protect—ask the student how they are and listen to their concerns
- Inform—inform the student and their family of any key messaging, including health and safety, child protection or educational messages
- Educate—reviewing the student’s learning during the past week, including progress through any learning packs, radio or text message content.

At the end of the call, the teacher and student agreed on the schoolwork to be completed over the next two weeks and scheduled the next phone call. In addition to calls, PEAS also sent SMS messages during school closure that also included material on safeguarding, positive discipline, study tips, Covid‐19 prevention, and notifications of radio lessons to be aired.

In contrast, the core aim of radio lessons was to ensure that students stayed connected with learning during the school closures; this provision was still punctuated with safeguarding and health messaging, and opportunities to lift students' spirits, such as singalongs, prayer and contemplation and family games. The radio lesson content was aligned to the Ugandan national curriculum and focused on core concepts in a range of subjects, including chemistry, biology, physics, mathematics, English, and agriculture. The radio show provided different kinds of assessment strategies such as tests, quizzes, and games so learners could assess their progress throughout the hour‐long lessons.

It is the relationship of these components to girls' academic, social, and emotional learning, as well as their demographic background, that will be specifically explored in this study. To this end, this article presents an analysis of a survey dataset which was collected, via phone calls to students, in November 2020. The survey dataset was collected by RDM (Research Development and Management)—a research and data management consultancy based in Uganda—on behalf of Jigsaw Consult, a UK‐based social enterprise. Jigsaw Consult commissioned the data collection as part of their independent endline evaluation of PEAS' “GEARRing Up for Success after School” programme (PEAS, 2021).^1^

By focusing upon EdTech use in the context of rural and low‐income schools within Uganda, the findings shed light upon EdTech use in an understudied context. The findings are of practical significance for educators in this particular context, but also in terms of how to reach the most marginalized in other education systems. The forms of EdTech used have been included in several countries' Covid‐19 emergency responses (Center for Global Development, 2021), so the findings contribute to the emergent evidence base in terms of robust understanding of the impact of the pandemic upon education. Beyond emergency responses, the focus upon gender means that the findings are also helpful in terms of the potential use of EdTech alongside formal schooling, in order to provide additional support to girls.

## 3 METHODOLOGY

A mixed‐methods sequential explanatory case‐study approach was used for the study (Creswell et al., 2003; Ivankova et al., 2006). Data collection and analysis comprised two phases: first, analysis of the student survey dataset; and second, an interview with PEAS staff to explore the survey findings.

The first phase involved analysis of a cross‐sectional student survey dataset (*N* = 483) of students in S4, S5, and S6 from 28 PEAS schools across Uganda. Enrolment in PEAS schools reflects a wider trend of decreasing proportions of girls enrolled in school in Uganda as grade increases (MoES, 2017). The data were collected in November 2020, via phone calls to students; it was estimated that 450 students (150 students per year group) would be sufficient to achieve adequate statistical power for the anticipated analyses.^2^ A mixture of stratified convenience and random sampling was used to select students. As the study primarily focused on girls, the sampling strategy involved reaching all female students in S5 and S6, with boys being contacted later, until the sample size of 150 students per year group was met. The dataset was not representative of PEAS' schools' population, and male students ultimately ended up being more strongly represented. The total PEAS schools sampling frame, and number of girls and boys included in the sample per grade, are shown in Table 1. Students ranged in age from 15 to 24 years old, with a median age of 18 years old for girls and women, and 19 years old for boys and men.

**Table 1:** Survey sample according to grade and gender

Grade and total student numbers	S4 (1440 boys, 1304 girls)	S5 (115 boys, 73 girls)	S6 (98 boys, 67 girls)	Total
Sample n boys	74	98	98	270
Sample n girls	85	61	67	213
Total	159	159	165	483

The survey data were analysed in R statistical software using chi‐square tests and logistic regressions. The key variables explored in this study relate to learning and the frequency of use of different forms of EdTech. Learning variables included two Likert‐type questions on students' self‐confidence, one on students' self‐esteem and one on students' perception of their academic learning progress during Covid‐19‐related school closures. While having an observed measure of academic learning (as opposed to students' perceptions) would have been preferable, it was not possible given Covid‐19 restrictions, time constraints, and the key evaluation aims of the programme at the time. The key variables exploring EdTech use were all single Likert‐type questions that asked students about their frequency of: (1) listening to PEAS' educational radio broadcasts; (2) receiving check‐up calls from their teachers; and (3) receiving SMS messages from their teachers. Students were also asked questions about demographic information and other potentially mediating factors, as well as questions related to EdTech use. Further, poverty was considered a control variable, in statistical analyses that allowed for multiple predictors, since it is a key determinant of student outcomes. Disaggregation, or the insertion of other variables as control variables, was generally not done because of sample size limitations. However, when significant differences were not found while exploring the key variables of interest, further exploration (such as with respect to class groupings) were considered.

While the survey analysis examined what differences exist in how different modes of EdTech are received by students, the second phase of data collection followed on to explore why those differences might exist, through a key informant interview with two PEAS' administrative staff during May 2021. One of the interviewees was PEAS' Chief Technical Officer (CTO), who had worked at PEAS for 8.5 years, including two years living and working for PEAS in Zambia and one year in Uganda. The CTO worked closely with Ugandan technical leads and the delivery team during the pandemic to collaboratively design and implement PEAS' remote education programme. The other interviewee was the Global Impact and Data Lead at PEAS for two years, working with the Uganda team on a daily basis. They provided support and oversight to the PEAS Uganda team in terms of monitoring, evaluation, and learning in relation to PEAS' programmes and reviewed and analysed student and school data. Both interviewees were based in the UK at the time of writing. The interview presented PEAS' staff with the findings of the quantitative analysis, and asked for their thoughts and explanations for the reasons behind the results. They were also asked questions about the PEAS' programme, and particularly interventions that were related to EdTech and girls' education.

Child protection and ethical considerations were prioritized during data collection. The enumerator team received detailed guidance and training on child protection and safeguarding in GEC‐T evaluations. The objectives of the study were explained to participants, and informed consent was required for the data collected. FCDO and PEAS policies regarding child protection and confidentiality were also adhered to.

While every effort has been made to ensure that the data were collected, analysed, and discussed in a fair and appropriate manner, limitations to the study should be acknowledged. Sample size limitations meant that further disaggregation, as well as more complex statistical models, could not be confidently explored. Additionally, some key variables were based on single‐item questions rather than on validated measures, and on perception rather than observation. The PEAS interviewees also noted that because of the Covid‐19 pandemic, PEAS was unable to host in‐person orientation with caregivers on EdTech distance learning, which may have reduced their efficacy. Finally, the authors' positionality was a limitation. None of the authors are Ugandan, or based in Uganda, and so they do not have lived understanding of the context and experiences of the study’s participants. Future studies can aim to involve more Ugandan authors and complement quantitative studies with in‐depth qualitative data from Ugandan women.

## 4 RESULTS AND DISCUSSION

The findings of the data analysis are discussed in this section. Each sub‐section reflects one of the four research questions in turn, presenting the results alongside a short discussion of the findings.

### 4.1 Challenges of using different types of EdTech according to gender

Girls engaged with the three types of EdTech to differing extents. Approximately half used radio, increasing to 73.2% for SMS messages and 81.2% for telephone calls (Table 2).

**Table 2:** Number of students who engaged with different types of EdTech at least once during the Covid‐19 school closures

		Radio	Telephone calls	SMS messages
Engaged with EdTech	Female	112 (52.6%)	173 (81.2%)	156 (73.2%)
	Male	138 (51.1%)	220 (81.5%)	187 (69.3%)
	Total	245 (50.7%)	393 (81.4%)	343 (71%)
Did not engage with EdTech	Female	101 (47.4%)	40 (18.8%)	57 (26.8%)
	Male	132 (48.9%)	50 (18.5%)	83 (30.7%)
	Total	233 (48.2%)	90 (18.6%)	140 (29%)

Notably, only 35 students (7.25% of all students) did not engage with any form of EdTech; there was a roughly equal split by gender. Students who had not engaged with EdTech were asked why they had not done so (students who did not engage with a form of EdTech were only allowed to select one reason why). Some students had not engaged simply because they had not received SMS messages (*n* = 45; 32.1%) or phone calls (*n* = 14; 15.6%) or were not in the broadcast range of radio programmes (*n* = 26; 11.2%). A lack of access to devices was also often reported: no access to radio (*n* = 85; 36.5%), no access to a phone for SMS messages (*n* = 37; 26.4%), and no access to a phone for calls (*n* = 25; 27.8%).

Some differences emerged along gender lines, although few were statistically significant (as evidenced by chi‐square tests). Female students (*n* = 9; 22.5%) were more likely than male students (*n* = 5; 10.0%) to report not having access to their caregivers' phones when teachers called. There were also significant differences in the frequency with which students spoke to their teacher on the phone (*X* ^*2*^ [2, 393] = 6.904, *p* = 0.032, *Φ*_*Cramer*_ = 0.133). The median boy and girl each spoke to their teachers monthly. However, only 17.7% of boys spoke to their teachers less than monthly, compared with 28.9% of girls.

The interview with PEAS' staff provided insight into this gendered difference. One contributing factor could be that some caregivers were reluctant to give their daughter access to their phone when the teacher calling them was male as a safeguarding measure (most secondary school teachers in rural Uganda are male). Similarly, female students more frequently reported (*n* = 7; 12.3%)—compared to male students (*n* = 5; 6.0%)—that their caregivers did not share SMS messages from their teachers with them. Although the sample here is small, it suggests that female students are twice as likely to be denied access and reflects some of the reduced access, and freedom to access, that girls often face (Webb et al., 2020), for reasons ranging from safeguarding (Zelezny‐Green, 2014) to social attitudes that hold that women are not as capable with technology as men (Vilakati, 2014; Yeba, 2012; Zelezny‐Green, 2011).

Students who *had* engaged with the radio programmes were also asked about the challenges that they faced in tuning in (students who had engaged with the radio programming were allowed to select multiple reasons for this). The most common challenge was that the time of the broadcast clashed with their domestic responsibilities (*n* = 147; 60.5%) or work (*n* = 76; 31.3%), with significant differences by gender. Male students were much more likely (70.4%) than female students (48.1%) to report domestic responsibilities as a challenge (*X* ^*2*^ [1, 243] = 12.398, *p* = 0.000, *Φ*_*Cramer*_ = 0.226). While this result may appear surprising, given the common narrative of female students having greater domestic responsibilities than male students, it may be masking the possibility that girls with the greatest domestic responsibilities may simply not be enrolled in school—gender parity in school enrolment in Uganda decreases from S4 through to S6 (MoES, 2017). So, it is still possible that girls have more domestic responsibilities.

Results from PEAS' internal monitoring also shows that both boys and girls have substantial amounts of domestic responsibilities, which presents the greatest challenge to regularly listening to radio programmes. However, they suggest that the kind of responsibilities are often divided along gender lines. Girls tend to have greater responsibilities in caring for family, and boys are often tasked with conducting errands outside the home and helping in gardens. It is therefore possible that the different kinds of chores mean the students are available at different times of day according to their gender, and the scheduling of radio programmes may happen to fit better around the types of domestic responsibilities often assigned to girls. It is also possible that caregivers allow girls breaks from their chores to tune into radio programmes—which might be seen as a safer route to education engagement than telephone calls with teachers. More research is needed to explore why.

Other gender divides also arose with regard to the experience of students who had engaged with radio programmes. Male students were more than twice as likely as female students to report lack of interest in the programmes (*X* ^*2*^ [1, 243] = 9.112, *p* = 0.003, *Φ*_*Cramer*_ = 0.194), that the lessons were too difficult (*X* ^*2*^ [1, 243] = 7.549, *p* = 0.006, *Φ*_*Cramer*_ = 0.176), and that the lessons were not interesting (*X* ^*2*^ [1, 243] = 6.013, *p* = 0.014, *Φ*_*Cramer*_ = 0.157). Female students were therefore significantly more engaged in radio than their male peers.

PEAS internal survey data shed some light on this finding. In December 2020 PEAS ran an internal phone survey with a small sample of students and caregivers to collect feedback from participants. This was used to help adapt and improve programme design and implementation, and suggested two possible reasons why female students were more engaged in radio than their male peers. First, more girls (76%) than boys (67%) reported receiving support from their caregiver with home study. More support from caregivers for girls' rather than boys' home study could be seen as a surprising finding, considering evidence to suggest that caregivers generally see boys' education as a better investment than girls' (Ministry of Gender, Labour and Social Development & UNICEF, 2015). However perhaps the unique circumstances of the Covid‐19 pandemic and prolonged school closure led to heightened levels of anxiety regarding girls' futures, and therefore led to a greater focus from caregivers on girls' education. This might have then translated into increased support for helping girls engage with the mode of remote education that caregivers saw as the least likely to expose girls to safeguarding risks: radio.

Higher levels of support for girls' home study than that of boys could also be connected to the nature of household chores that boys are more frequently responsible for than girls, which often involve work outside the household. If caregivers are reliant on boys' support for these types of tasks, they may be less inclined to encourage them to stay at home to participate in the radio lessons. The internal PEAS survey also found much higher proportions of girls than boys reported needing support with STEM subjects, which were the focus of the radio shows (in addition to English and agriculture). If girls were feeling more anxious than boys about these particular subjects, this could have led to higher levels of motivation among girls for engaging with STEM focused radio lessons.

### 4.2 Factors and their relationship to the perceived usefulness of EdTech

To address this research question, the analysis drew upon data from outcome variables (survey response items) and a range of potential predictors, including parents' education, frequency of chores, age, gender, and Poverty Probability Index (PPI) scores (to measure wealth). The average scores of the outcome variables, by gender, are summarized in the Appendix (Table A1).

Table A1 shows little overall difference in how helpful male and female students found EdTech (with the exception of radio). However, Table A1 presents only an overview, and the data were explored in further detail using proportional odds logistic regressions, which showed that there are clear differences in how different predictors relate to those outcomes. The following model was used for the regression analysis:

$$\text{logit}(P\left(Y\leq j\right)=\text{βj0}–\eta 1\text{χ1}–\ldots –\text{ηpχp}$$

where (P(Y ≤ j) refers to the probability of students' either finding radio, SMS messages, or telephone calls helpful and/or motivational less than or equal to a specific category, *β*j refers to Y intercept and *η* refers to the log odds of believing that EdTech’s helpfulness was related to a specific predictor: *χ*, in this case, the mediating factors of Parents’ Education, Frequency of Chores, Age, Gender, and PPI scores (wealth).

All mediating factors were included in each regression (unless otherwise stated) and regressed onto the respective EdTech outcome variable. Results will be presented in turn by the type of EdTech.

#### 4.2.1 Radio

Results show that most of the mediating factors considered had no meaningful effect on whether students found radio lessons helpful for their learning. The exception to this was the number of chores that students had to do. Students who had more chores appeared slightly less likely to find the radio programmes helpful for their learning (*β* = −0.174, *t* = −1.918, *p* = 0.055). However, in follow‐up regressions, where the data were disaggregated by gender, the strength and significance of the “chores” effect only held for boys (though marginally so: *β* = −0.224, *t* = −1.736, *p* = 0.083). This echoes the previous discussion, which suggested that chores appear to be particularly limiting for boys—at least in terms of learning via the radio lessons.

#### 4.2.2 SMS messages

The formal education of students' parents was the only mediating factor significantly related to whether students found the information provided in SMS messages useful for keeping them safe. The odds of students with more highly educated parents being more likely to find the messages helpful were 1.32 times greater than for students with less well‐educated parents (*t* = 3.102, *p* = 0.002). When the data were disaggregated by gender, the effect was weaker and only marginally significant for boys. The interview with PEAS' staff suggested a possible explanation for this, noting that parents who do not understand the language of the SMS messages—English—are likely to have less formal education and are also less likely to pass the messages on to their children. Furthermore, PEAS staff suggested that people on Ugandan mobile networks often receive frequent spam messages. It is possible that parents with less formal education, and therefore weaker English language skills, dismiss the educational SMS messages as spam.

These findings underscore the importance of considering and employing commonly spoken local languages—as opposed to only “colonial” languages'—in education (Akyeampong et al., 2018; Altinyelken et al., 2014; Bunyi, 1999; Damani, 2020). Although English is widely considered as the language of formal education in Uganda, and can be a useful language for students (and caregivers) to learn, using it to the exclusion of local languages may cause inequity in children’s access to education through EdTech (Kirunda & Ogavu, 2015). It is also worth considering that more formally educated parents, having received a similar education themselves, may also be better able to facilitate their children’s’ engagement with educational material, regardless of English language proficiency (Kakuba et al., 2021). This aligns with PEAS’ observations from internal surveys with parents and caregivers: among caregivers that reported not supporting their child with studying at home, the most common reason was that they do not feel they have the knowledge or skills necessary to do so.

Another regression explored whether the SMS messages inspired and motivated students to participate in educational activities. Students who had more chores to complete were significantly less likely (*β* = −0.176, *t* = −2.423, *p* = 0.015) to find the SMS messages inspiring. No other mediating factor had any significant relationship relating to finding the messages inspiring and motivational; again, parents' education had a positive effect (albeit insignificant: *β* = 0.974, *t* = 1.24, *p* = 0.215).

Disaggregating the data by gender provided further insight. While having more chores (*β* = −0.299, *t* = −2.753, *p* = 0.006) was associated with girls being significantly less able to take inspiration from SMS messages, this was not the case for boys (*β* = −0.045, *t* = −0.448, *p* = 0.654). This is worth further exploration considering the previously discussed findings concerning radio, which consistently suggested that chores were a barrier for boys' engagement. While girls may be able to engage with radio lessons—possibly because their typically allocated chores may be domestic, as opposed to boys' chores which may be outside the home, or due to caregivers potentially allowing girls a break from chores to tune into broadcasts—the same cannot be said for SMS messages. As suggested previously, this may be related to safeguarding issues that caregivers have concerning girls' phone use. Further research is needed to understand exactly why.

The gender disaggregation revealed further nuance. While both wealthier and poorer girls took similar inspiration from SMS messages (*β* = 0.002, *t* = 0.212, *p* = 0.832), poorer boys were slightly, though significantly, more likely to take inspiration from the SMS messages than wealthier boys (*β* = −0.026, *t* = −2.176, *p* = 0.03). A separate regression to explore the interaction between gender and poverty on finding SMS messages inspiring—with no other control variables in the equation—showed gender to have a significant interaction with poverty (*β* = −0.032, *t* = −1.965, *p* = 0.0494). Examples in the literature review showed that girls may disproportionately benefit from EdTech when they are afforded access to it, but this finding may point to something broader: that marginalized students, when afforded access to EdTech, have the potential to find even greater educational inspiration through it than less marginalized students.

Poor students face several barriers to participation in secondary school; poverty can create pressure for young people to become economically active to support themselves and their family, and can also mean that young people are unable to afford school fees. An awareness of the challenges they face could perhaps lead to higher levels of anxiety about being able to return to school, which may then mean that poorer boys find the SMS messages (which were intended by PEAS to be uplifting) more meaningful. Poorer boys are also less likely to have opportunities to engage with other educational activities beyond the PEAS interventions. It is possible that marginally wealthier boys could access other forms of educational support, such as education supplements in local newspapers, which could perhaps have diluted the effect of specific PEAS interventions. This result does not demonstrate a large effect, but it hints at where more in‐depth research might be beneficial.

#### 4.2.3 Telephone calls

There was no relationship between any of the mediating factors and the effects of calls in relation to wellbeing and helping understand how to look after themselves. However, significant effects were found as to whether students found the phone calls helpful for self‐study. In descending order of effect size: students who had more chores felt they were less likely to benefit from teachers' help with self‐study (*β* = −0.145, *t* = −2.185, *p* = 0.029); students from households with more highly educated caregivers were more likely to believe that they could discuss their learning with teachers (*β* = 0.131, *t* = 1.919, *p* = 0.053); and poorer students were more likely to feel that they were able to discuss their learning with teachers on the phone (*β* = −0.023, *t* = −2.945, *p* = 0.003). These results echo some of the earlier findings for students' engagement with SMS messages, and some of the same discussion points will apply. Formally educated parents may be better able/more likely to facilitate their children’s engagement with technology, however when children who are more marginalized (less wealthy in this case) do have access to technology, they may benefit to a greater extent. Notably, these results do seem somewhat contradictory, since greater education tends to be related with greater wealth. However, it does also suggest that wealth does not wholly determine the extent to which children can benefit from technology. Further research is necessary to tease out the conditions under which wealth and parents’ education are of unique benefit.

Key differences in the results were observed when the data were disaggregated by gender. The effect of caregivers' education (*β* = 0.245, *t* = 2.503, *p* = 0.012) and poverty (*β* = −0.027, *t* = −2.623, *p* = 0.008) only held for boys and there was a marginal effect of chores for girls (*β* = −0.172, *t* = −1.755, *p* = 0.079). As discussed previously, it appears to be the case that, in contrast to the situation relating to radio, caregivers may not be allowing their female charges freedom to use phones for educational activities. However, poorer boys, especially those whose parents have some formal education, appear to find EdTech disproportionately more helpful for their learning than wealthier boys—again, a somewhat contradictory finding, considering the relationship typically assumed between wealth and higher levels of education, but one which suggests that wealth does not wholly determine the extent to which a student can benefit from teacher support with self‐study via EdTech. More research would be beneficial in this area.

### 4.3 EdTech modalities and their benefits for girls' and boys' perceived learning progress at home

Proportional odds logistic regressions were used to answer this research question. The data were first explored using all participants, before focusing upon girls. All three modes of EdTech were entered into each regression equation, alongside poverty, with perceived learning progress during the pandemic being the outcome variable.

Telephone calls appeared to be the most beneficial for students' perceived learning progress. The odds of students believing they had progressed more in their learning during the pandemic was 1.38 times that of students who did not receive telephone calls (*β* = 0.320, *t* = 3.523, *p* < 0.001). SMS messages (*β* = 0.255, *t* = 3.274, *p* = 0.001) and radio (*β* = 0.113, *t* = 1.988, *p* = 0.047) were also found to have similarly small, but positive and significant, relationships with the perceived level of learning progress.

However, when the students were disaggregated by gender, slightly different results were found. Radio (*β* = 0.219, *t* = 2.490, *p* = 0.012) had a significant effect on girls' perceived learning progress at home, and SMS messages marginally so (*β* = 0.248, *t* = 1.922, *p* = 0.055). Conversely, telephone calls (*β* = 0.378, *t* = 3.208, *p* = 0.001) and SMS messages (*β* = 0.267, *t* = 2.714, *p* = 0.007) appeared to have small, but positive and significant, effects on boys' perceived learning progress. This suggests that EdTech played a positive role in students' perceived learning progress during the Covid‐19 school closures—regardless of whether students were wealthy or poor. No form of EdTech stands out as having a particularly large effect on perceived learning progress though, and so confidently stating that one EdTech is more beneficial than another is problematic. However, the differences by gender are still noteworthy.

Radio and SMS messages appear to be particularly important in girls' remote education. Previously discussed findings have already suggested reasons for why radio might be important for reaching girls, including caregivers' potential safeguarding concerns, girls' reduced freedom to use hi‐tech, as well as girls being at home and able to listen to radio despite chores. Boys, however, may have chores away from home and be allowed more freedom to use phones. As such, a lack of consistent access to a phone may hinder girls' ability to take the fullest advantage of telephone calls from teachers. Indeed, boys (*M* = 1.74, *SD* = 1.04) reported engaging with telephone calls slightly more often than girls (*M* = 1.61, *SD* = 1.03) did.

Although not a focus intervention of this study, students were also given paper‐based learning packs. When included in the regression analyses, these packs were more “impactful” on girls' education than all forms of EdTech, but were less effective than EdTech (except radio) for boys.

The interview with PEAS' staff highlighted that SMS, telephone calls, and radio listening were not mandatory for students. The EdTech activities explored may have self‐selected the students most motivated or most able to study, which in turn may have made them more likely to have a stronger perceived learning progress.

### 4.4 EdTech modalities and their perceived benefits for girls' and boys' self‐esteem and self‐confidence development

The data were examined using binomial logistic regressions since the relevant outcome variables were each almost exclusively clustered in two ordered categories. Each type of EdTech, and poverty, were entered into the equation as “predictors.” Students' self‐esteem, their confidence in success at school, and their confidence in success beyond school were each outcomes in unique regressions.

There was no significant relationship between engagement with any form of EdTech and self‐esteem. However, there appeared to be significant positive effects of EdTech on students' confidence. The youngest cohort of students (those in S4) appeared to benefit substantially from receiving phone calls from teachers. The odds of S4 students having more confidence in their ability to succeed at school was 3.11 times that of students who had not received phone calls (*z* = 1.999, *p* < 0.05) from their teachers. There were no significant effects for older students, or differences based on gender.

When students' confidence in their prospects of success beyond school was examined, telephone calls again appeared to have a positive effect (*β* = 0.564, *z* = 1.988, *p* = 0.047). However, further examination revealed this effect to, again, only be significant for younger students (those in S4 and S5). This suggests that EdTech, and particularly speaking one‐on‐one with teachers over the phone, can have some small and positive effect on students' SEL.

That telephone calls, but not radio, had some effect on SEL may be intentional. While all EdTech interventions in some way aimed to boost students' learning during the Covid‐19‐related school closures, the phone calls and SMS messages more explicitly aimed to improve wellbeing and SEL. The PEAS interviewees noted that PEAS teachers provided education and pastoral support remotely to students through the telephone calls. Each teacher was responsible for calling their own class of students once a fortnight to cover health, safeguarding, and education topics, using guidance and content shared by the PEAS programme leads. The most vulnerable students, for example those who are at higher risk of abuse or pregnancy, received more frequent phone calls.

Given the differences in content and design of radio and SMS components of the programme (outlined earlier in the “introduction to PEAS” section), while it is not surprising that no effect was found for radio, it is perhaps surprising as regards SMS messages. One potential reason for this difference may be that telephone calls allow more interaction than SMS messages. It may be the case that interaction is key if EdTech interventions are to help improve wellbeing and SEL (Allen et al., 2011). However, it is still unclear why only younger students appeared to benefit in terms of their wellbeing and SEL. Further research is still necessary to explore this and how students' SEL might be served by different forms of EdTech.

## 5 CONCLUSION AND IMPLICATIONS

The results have implications for practice and policies focused on girls' education through technology, both during periods of emergency school closure and also potentially to support girls' education alongside formal schooling. Recommendations include: (1) ensuring that caregivers are involved in the strategies developed for girls' education, as well as girls' education at home; (2) designing interactivity into the use of EdTech; (3) utilizing different forms of EdTech to reach diverse populations; and (4) considering language.

### 5.1 Supporting caregivers at home

The results highlight the importance of caregivers' involvement if students are to benefit from technology‐mediated distance education. More highly educated parents were better able to support girls in their learning; therefore, focus should also be on ways in which caregivers can be supported in their role, through provision of sessions about how to help children to study, for example. In arranging such sessions, however, care must be taken to do so in ways which allow for flexibility around caregivers' other commitments and reduce the risk of exacerbating the effects of inequalities. Further, as girls' access to phones appeared to be limited because of safeguarding concerns and social attitudes against girls' technology use, it is key that caregivers be afforded support on how they might help ensure girls' safety while using technology. Consulting caregivers when devising strategies for girls' EdTech use, and participatory design, may help ensure that girls are given more freedom to access digital learning.

### 5.2 Combining EdTech with human interaction

The analysis showed the potential of SMS text messages and phone calls to boost students' confidence and belief in their ability to succeed. This finding, along with PEAS' own observations, points to the value of human interaction alongside other low‐tech channels to provide education content to students, to reassure students, check on their wellbeing and listen to their concerns. Individual phone calls from teachers to students and caregivers may be challenging to implement system‐wide, but could be used as a targeted tool, for example to support students who are low in confidence, falling behind, or at risk of dropping out. Alongside other forms of EdTech, phone calls could be used to help caregivers and girls to engage with and access other channels (e.g. getting caregiver buy‐in for girls having access to their phone to access educational SMS text messages).

### 5.3 Reaching different demographic groups of students through different EdTech

The findings underscore the importance of recognizing the potential role that low‐cost technologies—such as radios, mobile phones, and paper‐based resource packs—can play in educational initiatives, and also the importance of considering the context of use. The analysis highlighted a number of ways that access to different forms of technology varied by demographic group: while girls were more likely to report a lack of access to mobile phones, they were significantly more engaged with radio learning programmes. Similarly, factors such as parents' education or boys' poverty were found to correspond with the usefulness of and inspiration taken from SMS messages. It is recommended that different forms of EdTech be utilized to reach diverse population groups, and that careful research be undertaken to determine the most appropriate approach in any given context.

### 5.4 Importance of considering language

The language of instruction is a key consideration for any EdTech initiative. The association found between a caregiver’s level of education and the extent to which students found SMS messages to be helpful, along with PEAS’ own observations, indicated that using English (or other “colonial” languages) to the exclusion of local languages may impede children’s access to the benefits of learning via EdTech.

### 5.5 Future research

This study contributes to the evidence gaps within girls' education in LMICs and the use of EdTech, and also identifies key areas which demand further research. Findings relating to age point to specific questions to follow‐up on, including exploring the association between gender and age, and the relationship between student age and EdTech. The finding that some of the interventions helped with SEL—for younger but not older students—suggests that further research would help understand when and why differences emerge, and which forms of technology support this. Further work is also needed to explore the specific barriers highlighted in the study, including unpacking the relationship between household responsibilities and gender, and a more nuanced understanding of caregivers' safeguarding concerns around girls' mobile phone use, in order to address their current role as “gatekeepers” to technology.

There are also broader questions in relation to the potential use of EdTech across educational systems at scale. This study only incorporated data from rural regions of Uganda, which raises a question around regional variation, and how EdTech use may differ, by gender, in urban and peri‐urban areas. The finding that learning packs had a significant impact on girls' learning points to the need for further comparative work around different forms of EdTech and low/no‐tech initiatives; a detailed cost–benefit analysis could be particularly useful. EdTech does appear to have great potential for rural Ugandan girls' academic, social, and emotional learning; future research can better explain how.

## AUTHOR CONTRIBUTION STATEMENT

Kalifa Damani, Rebecca Daltry, and Katy Jordan conducted the interviews, analysed the data and wrote the article. Libby Hills and Laura Evans are PEAS staff representatives who were given the opportunity to review the article before it was submitted for publication. Some of their comments and suggestions were incorporated into the article at the discretion of the first three authors to ensure independence of the findings.

## FUNDING

We gratefully acknowledge funding provided to support PEAS and the evaluation from the Foreign, Commonwealth and Development Office (FCDO), under the Girls Education Challenge Transition fund (GEC‐T), and other PEAS funders. We also thank the EdTech Hub (http://www.edtechhub.org) for support during writing up.

## Data Availability

The data that support the findings of this study are available on request from the corresponding author. The data are not publicly available due to privacy or ethical restrictions.

## Footnotes

^1^
One of the authors of this article, Kalifa Damani, contributed to the survey design and data collection.

^2^
Notably, although p < 0.05 was the benchmark for significance, “marginal significance” was noted in places where p < 0.1.

## 1

**Table A1:** Average score of outcome variables, by gender

Variable	Gender	Averages
Radio is helpful for learning	Female	*M* = 4.44, *SD* = 0.856 *Mdn* = 5 (Strongly Agree) *n* = 109
	Male	*M* = 4.3, *SD* = 0.864 *Mdn* = 4 (Agree) *n* = 132
SMS is helpful for safeguarding	Female	*M* = 4.61, *SD* = 0.679 *Mdn* = 5 (Strongly agree) *n* = 155
	Male	*M* = 4.6, *SD* = 0.543 *Mdn* = 5 (Strongly agree) *n* = 187
SMS messages inspire and motivate	Female	*M* = 4.34, *SD* = 0.83 *Mdn* = 4 (Agree) *n* = 156
	Male	*M* = 4.29, *SD* = 0.911 *Mdn* = 4 (Agree) *n* = 187
Teachers spoke to me about my wellbeing on the phone	Female	*M* = 4.46, *SD* = 0.634 *Mdn* = 5 (Strongly Agree) *n* = 173
	Male	*M* = 4.454, *SD* = 0.73 *Mdn* = 5 (Strongly Agree) *n* = 220
Teachers discussed my self‐study with me	Female	*M* = 3.924, *SD* = 1.181 *Mdn* = 4 (Agree) *n* = 173
	Male	*M* = 4, *SD* = 1.106 *Mdn* = 4 (Agree) *n* = 220
Teachers inspire	Female	*M* = 4.4, *SD* = 0.697 *Mdn* = 4 (Agree) *n* = 173
	Male	*M* = 4.35, *SD* = 0.71 *Mdn* = 4 (Agree) *n* = 220
